# Good Manufacturing Practice-Compliant Cryopreserved and Thawed Native Adipose Tissue Ready for Fat Grafting

**DOI:** 10.3390/jcm13113028

**Published:** 2024-05-21

**Authors:** Giulio Rusconi, Martina Cremona, Matteo Gallazzi, Luca Mariotta, Mauro Gola, Eugenio Gandolfi, Matteo Malacco, Gianni Soldati

**Affiliations:** 1 Swiss Stem Cell Foundation, 6900 Lugano, Switzerlandmartina.cremona@sscf.ch (M.C.);; 2 Swiss Stem Cells Biotech AG, 8008 Zurich, Switzerland; 3 Clinica Sant’Anna, Swiss Medical Network, 6924 Sorengo, Switzerland

**Keywords:** adipose-derived stem cells, cryopreservation, adipose tissue

## Abstract

**Background**: As adipose tissue-derived mesenchymal stem cells are becoming the tool of choice for many clinical applications; standardized cryopreservation protocols are necessary to deliver high-quality samples. For this purpose, the cryopreservation and thawing of native adipose tissue under GMP conditions could represent an extremely useful and powerful tool for the direct reinfusion of the tissue, and consequently, of its stromal vascular fraction. **Methods**: In this study, 19 samples of adipose tissue were cryopreserved and characterized before and after storage in liquid nitrogen vapors. Of these 19 samples, 14 were processed in research and 5 in a GMP-compliant environment. Storage with and without cryopreservation medium was also evaluated. After one week to three months of storage, samples were thawed, washed, enzymatically digested, and characterized with flow cytometry. **Results**: The results show that there is a loss of nearly 50% of total nucleated cells during the cryopreservation/thawing process. Non-GMP and GMP samples are comparable for all parameters analyzed. This study also allowed us to exclude the cryopreservation of adipose tissue without any cryopreservation medium. **Conclusions**: The data shown in this work are consistent with the idea that native adipose tissue, if properly processed and controlled, could be a useful source of cells for regenerative medicine, keeping in mind that there is a clear difference in the quality between fresh and thawed samples.

## 1. Introduction

The enhancement of the innate healing response represents the goal of stem cell-based medicine, via autologous and allogeneic transplants, using native or bioengineered stem cells. Mesenchymal stem cells (MSCs) are found in several tissues in humans, whose role and importance has been re-evaluated due to their wide endocrine crosstalk potential [1]; in particular, adipose-derived stem cells (ASCs) were identified in adipose tissue (AT). In several studies, many groups found that adult stem cells derived from white AT potentially differentiate into different cell subtypes, making MSCs the major candidate for several applications in regenerative medicine and tissue engineering [2,3]. The use of fat grafting for reconstructive purposes is increasingly widespread among plastic surgeons, requiring the development of a cryopreservation protocol for lipoaspirate to avoid further liposuctions as well as to standardize the thawed lipoaspirate product. It has been demonstrated that adipose tissue is a great source of different cell types, such as endothelial cells, pericytes, and adipose and mesenchymal stem cells, and since it can easily be handled, adipose tissue collection and cryopreservation represent powerful tools for cosmetic autologous purposes, regenerative medicine, as well as cellular therapies [4]. Therefore, an optimal freezing strategy for native AT storage must be found to ensure long-life preservation for the subsequent repair, substitution, or even regeneration of damaged tissues [5,6,7,8]. The optimization of the freezing protocol required detailed studies to completely guarantee the long-lasting preservation of cell integrity, using balanced cryoprotective molecules, namely cryoprotectant agents (CPAs), such as di-methyl-sulfoxide (DMSO), due to their abilities to inhibit protein denaturation and avoid water crystallization in freezing cells [9,10,11]. Nevertheless, high concentrations of CPAs were demonstrated to induce several cellular lesions and toxic effects; therefore, the reduction in cellular exposure to CPAs at room temperature during freezing processes was found to limit the occurrence of toxic effects from cells [3]. To date, the cryopreservation of native AT and its application has been examined by few groups. Shoshani and colleagues proved a good level of AT survival in vivo after being injected in nude mice after 2 weeks of storage at −18 °C compared to those injected with non-frozen tissue [12]. Moreover, McRae’s group considered direct fat tissue preservation into −196 °C liquid nitrogen for 8 days and demonstrated the good preservation of mitochondrial metabolic activity after being grafted in vivo [13]. In both experiments, AT samples were frozen without any CPA addition. However, other studies reported the crucial role of CPAs to better preserve and maintain the high viability of tissues after thawing [14]. The direct application of thawed lipoaspirate on patients was also tested in 2006: Butterwick injected both hands of a clinical patient with a specific dose of fresh and thawed AT. He observed an equal fat increase in aging hands 1, 3, and 5 months after the injection using both frozen and fresh lipoaspirate [15].

The aim of this study was to analyze whether directly frozen native adipose tissue could be used, as already mentioned, in regenerative medicine. To this purpose, we analyzed both fresh and frozen samples processed in a GMP and non-GMP environment, stored with cryopreservation medium.

## 2. Materials and Methods

### 2.1. Adipose Tissue Collection

Liposuctions were performed in Switzerland between 2022 and 2023 by accredited plastic surgeons, following total or local anesthesia, with the surgical removal of 150 mL of adipose tissue. The adipose tissue removal was performed using 25 cm and 50 cm cannulas which were 4 mm in diameter with 2 mm aspiration holes. The Pal Microaire technique was used with 100% movement speed and suction power. The collected tissue was left for 45 min in sterile syringes in a vertical position to allow the separation of the aqueous, lower phase (containing blood and Klein’s solution of 500 mL of saline solution + 10 mL of lidocaine 2%) which was discarded. A total of 19 samples (14 processed in a non-GMP environment and 5 in a GMP-compliant environment) from 19 different healthy patients (range 20–77 years) were included. Five GMP-processed samples were screened for HIV 1 and 2 (Human Immunodeficiency Virus), HCV (Hepatitis C Virus), HBV (Hepatitis B Virus), and Treponema pallidum. Syringes were delivered in sterile bags to our research lab or the GMP clean room inside a certified transportation box at RT (20 ± 10 °C) and processed within 24 h from collection.

### 2.2. Adipose Tissue Transport

Lipoaspirate delivery displays the crucial step to guarantee the standardization of whole-tissue processing and storage. Samples were transported at RT in a temperature-controlled box, equipped with a temperature-recording probe (Escort dataloggers, Aesch bei Birmensdorf, Switzerland) to keep the temperature controlled every 15 min throughout the delivery (Figure S1). The temperature curve was downloaded and printed upon receipt of the kit. The package validation was performed by keeping it at 4 ± 2 °C and 33 ± 3 °C for 24 h, while temperatures were recorded by dataloggers. Subsequently, the collected lipoaspirate was divided into 3 different 50 mL syringes in the surgery room and syringes packed into the kit. The sealed kit was then transported via a dedicated transport carrier (Swissconnect AG, Luzern, Switzerland) to the lab or clean room. 

The validation of transportation was performed as follows: three different lipoaspirate samples were used, half of which were processed at two different time points, 24 and 48 h. 

### 2.3. Adipose Tissue Processing

Stromal vascular fraction (SVF) extraction was performed following our previously described protocol [16], using 30 mL Luer-Lock syringes (Henke-ject, Henke Sass Wolf, Tuttlingen, Germany) as separation funnels. In total, 10 mL of lipoaspirate was washed twice with 7 mL of Dulbecco’s Phosphate-Buffered Saline with calcium and magnesium (DPBS +/+, Merck, Darmstadt, Germany), with the syringe being held in a vertical position to allow the separation of AT cellular components from the aqueous phase, which was subsequently discarded. Then, the lipoaspirate was enzymatically digested through the enzyme mixture Celase^®^ (Cytori Therapeutics, San Diego, CA, USA), diluted in DPBS +/+ to a final concentration of 0.28 Wünsch U/mL, for 45 min at 37 °C under agitation, and the enzymatic reaction was stopped with 7 mL of DPBS without calcium and magnesium (DPBS −/−, Merck, Darmstadt, Germany), supplemented with 1% human albumin. The syringe was positioned in a vertical position and the lower aqueous phase, containing the SVF, was collected in a conical 50 mL tube (Corning, Tamaulipas, Mexico). Last two passages were performed twice to increase cell yield. The collection tubes were centrifuged for 5 min at 400 RCF and the pellet was then resuspended in 10 mL of DPBS −/− with 1% human albumin, following serial filtration with 100 μm and 40 μm filters (Corning Inc., New York, NY, USA). Finally, the hydrophilic phase containing the SVF was centrifuged at 400 RCF for 5 min and the resulting pellet was resuspended in 5% human albumin solution (CSL Behring, Bern, Switzerland).

### 2.4. Freezing Conditions Test and Cryopreservation of Native Adipose Tissue

We evaluated different conditions for the optimal cryopreservation of the adipose tissue, derived from 15 lipoaspirates. In total, 11 lipoaspirates were divided into two equal volumes of 10 mL each, with the first part being immediately processed for SVF extraction, whereas the remaining 10 mL was mixed in a 1:1 ratio with cryopreservation medium (CM) and dispensed in a 25 mL cryopreservation bag (Advatis, Assago, Italy). Cryopreservation bags were then sealed and frozen in a controlled-rate freezing machine (Ice-Cube, SY- LAB, Pukersdorf, Austria) with the following freezing program: from 4 °C to 0 °C in 6 min, then hold for 15 min at 0 °C. From 0 °C to −2 °C in 9 min and then hold for 2 min at −2 °C. From −2 °C to −35 °C in 1 min, from −35 °C to −15 °C in 4.5 min, and back to −35 °C in 20 min. Finally, the temperature was lowered from −35 °C to −100 °C in 13 min. Samples were then stored in the liquid nitrogen gas phase at ≤150 °C. To test freezing without any CM, the last 4 samples were divided into three volumes of 10 mL each and, in addition to immediate SVF extraction and storage with CM, an additional 10 mL was cryopreserved without CM. CM was prepared by mixing 40% Cryo-Sure DEX40 (WAK-Chemie Medical GmbH, Steinbach, Germany), 40% MEM alpha no phenol red (Merck, Darmstadt, Germany) and 20% human albumin 5% solution (CSL Behring, Bern, Switzerland), and it was maintained at 4 °C until use. 

### 2.5. Thawing of Cryopreserved Adipose Tissue

Samples were thawed from one week up to three months of storage and subsequently characterized. Cryopreservation bags were heated at 37 °C for 180 ± 15 s until a small ice cube was still visible. The sample was immediately transferred to a 50 mL syringe (BD, Heidelberg, Germany), mixed with 25 mL of cold DPBS +/+, and held in a vertical position at 4 °C to allow the separation of the tissue from the freezing medium to be discarded. A second washing step was performed to completely remove the freezing medium. The tissue was then enzymatically digested as described in Section 2.3.

### 2.6. SVF Characterization

The number of total nucleated cells (TNCs) and the viability percentage were determined with a Nucleocounter^®^ NC-100™ (Chemometec, Allerod, Denmark). Moreover, cells were characterized through multicolor flow cytometry analysis using a 10-channel Navios cytometer (Beckman Coulter, Nyon, Switzerland), and data were analyzed with Kaluza Software 1.2 (Beckmann Coulter, Nyon, Switzerland), as previously reported [17]. Briefly, 5.0 × 10^5^ cells were centrifuged 5 min at 400 RCF and the pellet was resuspended in 220 μL of DPBS −/− (Merck, Darmstadt, Germany) with 1% human AB serum (Pan-Biotech, Aidenbach, Germany). Subsequently, 100 μL of cell suspension was stained with Syto™ 40 (Thermo Fisher Inc., Waltham, MA, USA) and 7-Amino-Actinomycin D (7-AAD, Beckmann Coulter, Nyon, Switzerland) into a customized test tube containing CD34-, CD146-, and CD45-specific monoclonal antibodies (DuraClone Mix, Beckmann Coulter, Nyon, Switzerland), following the manufacturer’s instructions. After 20 min of incubation, erythrocytes were lysed with 1 mL of VersaLyse Lysing Solution (Beckmann Coulter, Nyon, Switzerland) for 15 min and samples were analyzed through multicolor flow cytometry.

### 2.7. SVF Characterization through Multicolor Flow Cytometry

Nucleated cells were detected using the nuclear marker Syto™ 40 to eliminate duplets and aggregates from the analysis, while 7-AAD staining was performed to discriminate viable cells. Among all the Viable Nucleated Cells (VNCs), the staining of the CD45 surface marker allowed the discrimination hematopoietic (CD45+) cells from non-hematopoietic (CD45−) cells. Through the subsequent CD146+ and CD34− surface marker staining, we were able to identify adipose-derived mesenchymal stem cells, namely ASCs, which display the CD45−CD34+CD146− phenotype, as well as CD45−CD34+CD146+ endothelial cells (ECs), pericytes, and precursor cells (CD45−CD34−CD146+) [17]. 

### 2.8. Colony-Forming Unit (CFU-F) Assay

To perform the CFU-F assay, 1000 cells were seeded in standard 6-well plates (Thermo Fisher Inc., Waltham, MA, USA) and cultured for 14 days in MEM Alpha Modification medium (Merck, Darmstadt, Germany) supplemented with 5% PLTGold^®^ human platelet lysate (Merck, Darmstadt, Germany) and incubated at 37 °C with 5% CO_2_. The culture medium was replaced every 3 days, and after 14 days of culture, cells were washed with DPBS, fixed with 10% formalin solution (Merck, Darmstadt, Germany) at RT for 15 min, and stained with a crystal violet solution (Merck, Darmstadt, Germany). After washing the plates with deionized water (Merck, Darmstadt, Germany), the number of colonies was counted. A colony consisting of more than 50 cells was defined as a CFU-F. 

### 2.9. Statistical Analyses

Statistical analyses were performed with Graph Pad Prism 8.4.2 (GraphPad Software, Boston, MA USA). For N > 2 cohort comparisons, nonparametric ANOVA (Kruskal–Wallis test) was used. The two-way ANOVA test was used to compare the cohorts of non-GMP vs. GMP groups. A non-parametric *t*-test (Mann–Whitney test) was used to compare *N* = 2 cohorts. *p*-values ≤ 0.05 indicated a statistically significant difference.

## 3. Results

**Figure 1:**
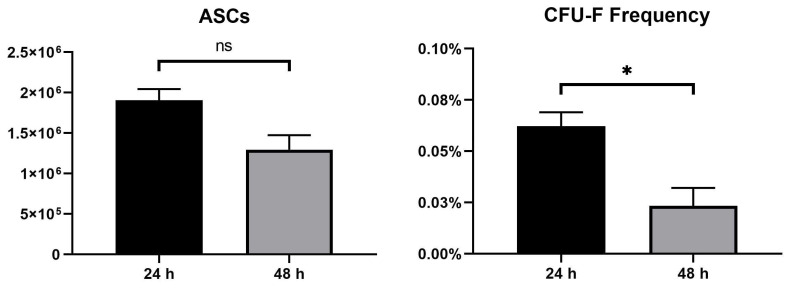
Lipoaspirate samples were transported and maintained at controlled temperature (+20 °C). SVF isolation was performed 24 and 48 h after surgery (*N* = 3). The cells were enumerated with hemocytometer and ASCs were characterized through flow cytometry. CFU-F frequency (%) was determined as follows: (n° of colonies/n° of inoculated cells)/100. * *p* < 0.05; ns = not significant.

We evaluated *N* = 19 native unprocessed AT samples to test the effect of direct cryopreservation without prior manipulations. In total, 14 samples were processed at the Swiss Stem Cell Foundation (SSCF) research laboratory and the remaining 5 were processed in a GMP-compliant environment (Class-A Isolators). We firstly developed and validated a dedicated kit to safely deliver samples from the surgery to the research lab and GMP facility, since it was demonstrated to maintain the temperature range of 20 ± 10 °C for 10 h when exposed to both low and high temperatures (Supplementary, Table S1). Moreover, we tested different delivery times in a pre-clinical set as described in Section 2.2 of the Materials and Methods. After processing the validation samples, we evaluated the potential ASC clonogenicity through the CFU-F assay, observing a decrease in cell yield, together with a reduced ASC yield (−31%) and consequent CFU-F frequency (−79%) in samples digested after 48 h from surgery, compared to those after 24 h (Figure 1). 

Based on these results, we set 24 h after surgery as the cutting edge for tissue digestion to better preserve and restore cellular functionalities. The transport was authorized by the Swiss regulatory body (Swissmedic, Bern, Switzerland).

We tested the lipoaspirate cryopreservation with and without cryomedium. After one week up to three months of storage, samples were thawed and enzymatically digested to extract and characterize the SVF. Consequently, we compared the cell number and viability of SVF cells extracted from both fresh and thawed native AT. 

**Figure 2:**
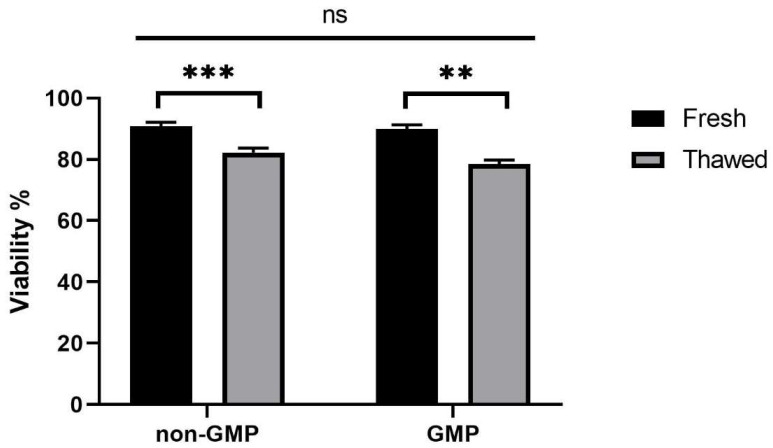
Cell viability of samples processed in non-GMP (*N* = 14) and in GMP-compliant environment (*N* = 5). ** *p* < 0.05; *** *p* < 0.001; ns = not significant, *p* ≥ 0.05.

Figure 2 shows data about cell viability on *N* = 14 non-GMP and *N* = 5 GMP-processed samples (GMP), comparing fresh and thawed AT. Although the statistical analysis revealed a significant difference among fresh and thawed samples (*p* < 0.001), their viability was still 82% (Figure 2). 

**Figure 3:**
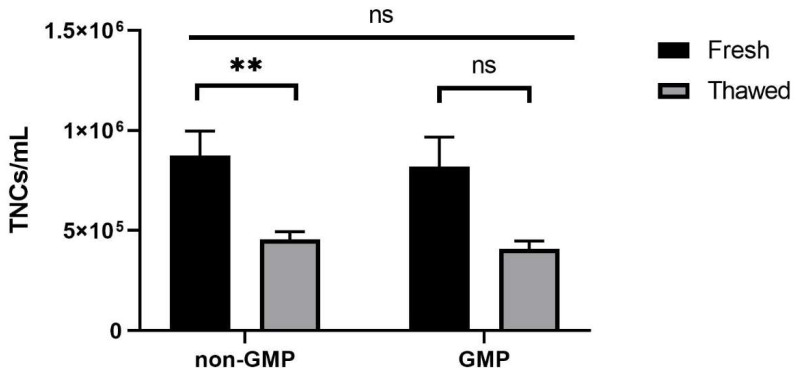
TNCs/mL of samples processed in non-GMP (*N* = 14) and in GMP-compliant environments (*N* = 5). ** *p* < 0.01; ns = not significant, *p* ≥ 0.05.

However, viability decreased significantly (*p* < 0.01) in samples stored without CM, lowering to 40% (Figure S2). By comparing the TNCs per mL of lipoaspirate, we observed a significant decrease in the number of cells in non-GMP samples frozen with CM (Figure 3, left side), decreasing from 8.75 × 10^5^ in fresh samples to 4.55 × 10^5^ TNCs/mL after thawing (*p* < 0.01). This significant reduction was even more evident in samples stored without CM, with 61% of cells lost throughout the process (TNCs/mL of 3.39 × 10^5^; *p* < 0.01, Figure S2); moreover, GMP processes highlighted a strong cell numerical decrease, with 47% of recovery in TNC number after cryopreservation with CM compared to the fresh samples (Figure 3, right). 

**Figure 4:**
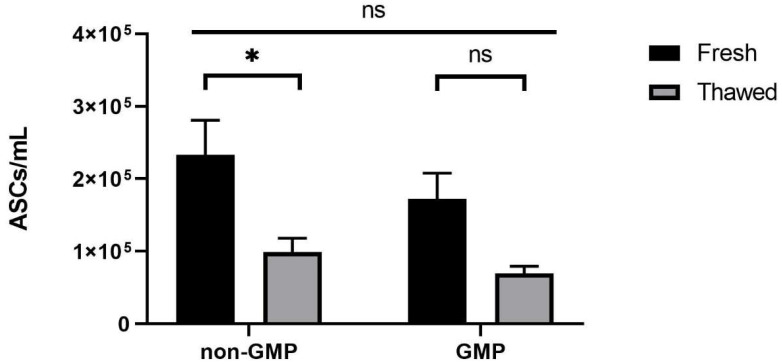
ASCs/mL of samples processed in non-GMP (*N* = 14) and in GMP-compliant environments (*N* = 5). * *p* < 0.05; ns = not significant, *p* ≥ 0.05.

However, no significant differences were observed among samples processed in both non-GMP and GMP environments. In particular, the number of ASCs changed significantly after freezing (Figure 4, left), since it was counted as 9.93 × 10^4^ per mL, significantly lower (*p* < 0.05) compared to fresh samples (2.33 × 10^5^). This loss was even higher in the no-CM group (*p* < 0.05), with 1.85 × 10^4^ ASCs/mL (Figure S2). 

**Figure 5:**
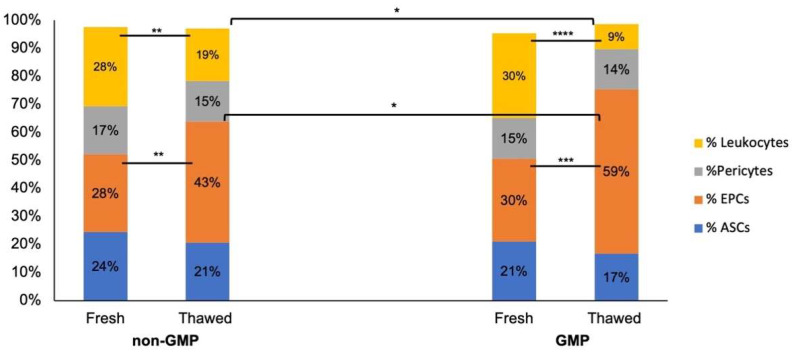
% Cellular sub-populations of samples processed in non-GMP (*N* = 14) and in GMP environments (*N* = 5). The brackets are relative to leukocyte %, fresh vs. thawed and EPC %, fresh vs. thawed in both non-GMP and GMP samples. * *p* < 0.05; ** *p* < 0.01; *** *p* < 0.001; **** *p* < 0.0001.

Altogether, these results led us to select native lipoaspirate storage in cryomedium. Regarding the concentration ratio (ASC/mL), the same numerical pauperization after cryopreservation was observed in GMP samples, with 6.95 × 10^4^ ASCs/mL after thawing (Figure 4, right), and no differences were observed among the two groups. The characterization of the heterogeneous cell population in the stromal vascular fraction (SVF) is shown in Figure 5. 

The comparison of cell population percentages among fresh and thawed samples in both non-GMP and GMP environments displayed a statistically significant difference in EPCs, increasing from 28% to 43% in the non-GMP group (*p* < 0.01) and from 30% to 59% in the GMP group (*p* < 0.001). Furthermore, the leukocyte frequency decreased from 28% to 19% in the non-GMP group (*p* < 0.01) and from 30% to 9% in the GMP group (*p* < 0.0001). The same comparison among different storage conditions (fresh, CM, and no-CM) in non-GMP samples for each cell population is shown in the Supplementary Material (Figures S3–S6). The frequency of cell populations in the three different conditions resulted in a decreased ASC percentage after cryopreservation with CM, while it was significantly lower (*p* < 0.01) in the no-CM samples. This significant pauperization was also observed in the leukocyte population in all three conditions (*p* < 0.0001), while pericytes significantly increased in the no-CM group compared to the fresh (*p* < 0.05) and CM groups (*p* < 0.01).

## 4. Discussion

Adipose tissue storage at low temperatures has been widely investigated, leading to conflictual results and conclusions over the years. This might be due, in part, to the lack of a standardized protocol for tissue processing and characterization, specifically for cell isolation and MSC identification, which lead to partial or missing parameters of analysis. Here, we provide, for the first time, a controlled study to better understand and use frozen and thawed native AT, where we study the cellular aspects of the thawed tissue compared to the same fresh sample processed in non-GMP and GMP conditions, which are crucial for the use of cell therapies in humans.

Different research groups have used the correct protocol to ensure better AT cryopreservation for future clinical use, which avoids tissue deterioration over the years and results in a consequent drop in recovery and unsuccessful autologous engraftment. In particular, the addition of DMSO as a CPA has been investigated since it affects the rate of water transport through the cell membrane, causing ice nucleation and crystal formation. However, due to its toxic potential at RT, the use of alternative CPAs such as trehalose (alone or in combination with DMSO) has been investigated with debated conclusions, and cryopreserved native AT in 0.5 M DMSO and 0.2 M trehalose was demonstrated to allow high cell viability after 48 to 72 h in culture after being thawed [18]. We reported the use of DMSO as a cryoprotectant agent for the freezing of native AT and compared the results to *N* = 4 samples cryopreserved without any CPA. Our findings show that the use of a cryoprotectant agent is needed to maximize the results in terms of the viability of cells. We observed that samples cryopreserved with DMSO indeed have higher viability (82%) when compared to samples stored without DMSO (40%). A recent study, where a similar cryopreservation protocol was used, showed how 67% of the volume of cryopreserved fat was recovered after thawing, with the observation of no histological differences between fresh and frozen tissue [4]. The same group analyzed different properties such as the phenotype, proliferation, and differentiation potential of expanded MSCs obtained after the enzymatic digestion of adipose tissue [19]. In this work, we did not analyze adipocyte cells nor expanded MSCs, since our focus was mainly on SVF cells.

Overall, we demonstrated that native lipoaspirate should be cryopreserved only with a specific cryoprotective agent such as DMSO and that the viability and total cell count of the isolated SVF were strongly reduced in the no-CM group by more than 50%, as also observed in AT cryopreserved in a commercial DMSO-free medium, where a similar loss of SVF cells with reduced viability was observed, when compared to AT cryopreserved with no CM [20], demonstrating the importance of DMSO as a cryoprotective agent. However, the addition of CM did not prevent cell loss during cryopreservation, maintaining, on the other hand, the viability of SVF cells above 80%, as already reported elsewhere [21]. We also reported a significant reduction in the cell count and viability of SVF cells after cryopreservation without CM, in line with previously published results [22]. The flow cytometry of SVF cells showed similar percentages of ASCs in fresh and thawed samples, whereas leukocytes were reduced and EPCs increased in the thawed samples. A recent study obtained similar results in terms of cell count, viability, and SVF sub-population percentages, using a CM with DMSO and human albumin [23]. In summary, we developed a standardized GMP protocol that could lead to the optimization of AT cryopreservation to guarantee a safer and more controlled quality of thawed native lipoaspirate, in order to use it further in reconstructive and cell-based treatments.

## Data Availability

All raw data are available upon request.

## Supplementary Materials

The following supporting information can be downloaded at https://www.mdpi.com/article/10.3390/jcm13113028/s1, Table S1: Validation of transportation box exposed to extreme temperatures; Figure S1: Example of datalogger temperature profile; Figure S2: Comparison of biological parameters between three different conditions in non-GMP samples. (A) Cell viability %; (B) TNCs/mL; (C) ASCs/mL. Fresh samples *N* = 14; thawed + CM *N* = 14; thawed no CM *N* = 4. * *p* < 0.05; ** *p* < 0.01; **** *p* < 0.0001. Figure S3: Comparison of ASCs % among fresh and thawed samples in non-GMP and GMP samples. (A) Non-GMP samples *N* = 14 for fresh and thawed + CM, *N* = 4 thawed no CM; (B) GMP samples *N* = 5. * *p* < 0.05; ** *p* < 0.01; ns = not significative; Figure S4: Comparison of EPCs % among fresh and thawed samples in non-GMP and GMP samples. (A) Non-GMP samples *N* = 14 for fresh and thawed + CM, *N* = 4 thawed no CM; (B) GMP samples *N* = 5. ** *p* < 0.01; ns = not significative. Figure S5: Comparison of leukocytes % among fresh and thawed samples in non-GMP and GMP samples. (A) Non-GMP samples *N* = 14 for fresh and thawed + CM, *N* = 4 thawed no CM. (B) GMP samples *N* = 5. ** *p* < 0.01; *** *p* < 0.001. Figure S6: Comparison of pericytes % among fresh and thawed in non-GMP and GMP samples. (A) Non-GMP samples *N* = 14 for fresh and thawed + CM, *N* = 4 thawed no CM; (B) GMP samples *N* = 5. * *p* < 0.05; ns = not significative, ≥0.05.
