# Supplementary material for: Good Manufacturing Practice-Compliant Cryopreserved and Thawed Native Adipose Tissue Ready for Fat Grafting
Source: J Clin Med. 2024 May 21;13(11):3028. doi: 10.3390/jcm13113028 (PMC11172459; doi:10.3390/jcm13113028)
Supplement: Supplementary file 1 [file jcm-13-03028-s001.zip › jcm-2980703-supplementary.pdf]

Supplementary Table

VALI-08

Transportbox gross	n=3	Datalogger Nr.	Date	Time to reach 10°C
SPEZ-036	Test 1	D-17	07.12.15	10:30
SPEZ-036	Test 2	D-11	07.12.15	10:30
SPEZ-036	Test 3	D-14	07.12.15	13:30
				11:30
Transportbox klein	n=3	Datalogger Nr.	Date	Time to reach 10°C
SPEZ-075	Test 1	D-13	04.12.15	11:00
SPEZ-075	Test 2	D-12	04.12.15	11:30
SPEZ-075	Test 3	D-19	07.12.15	10:00

Transportbox gross	n=3	Datalogger Nr.	Date	Time to reach 30°C
SPEZ-036	Test 1	D-18	27.11.15	15:00
SPEZ-036	Test 2	D-14	27.11.15	13:00
SPEZ-036	Test 3	D-13	27.11.15	15:00
Transportbox klein	n=3	Datalogger Nr.	Date	Time to reach 30°C
SPEZ-075	Test 1	D-12	27.11.15	14:40
SPEZ-075	Test 2	D-19	27.11.15	14:30
SPEZ-075	Test 3	D-17	04.12.15	14:30

Table S1. Validation of Transportation box exposed to extreme temperatures.

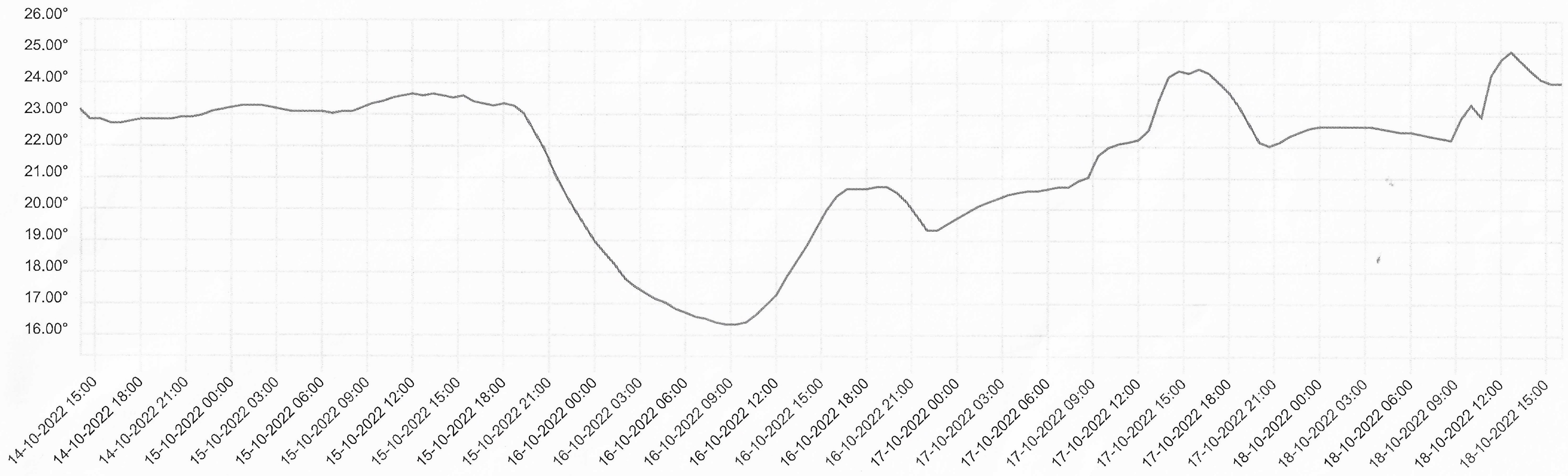

Figure S1. Example of Datalogger temperature profile.

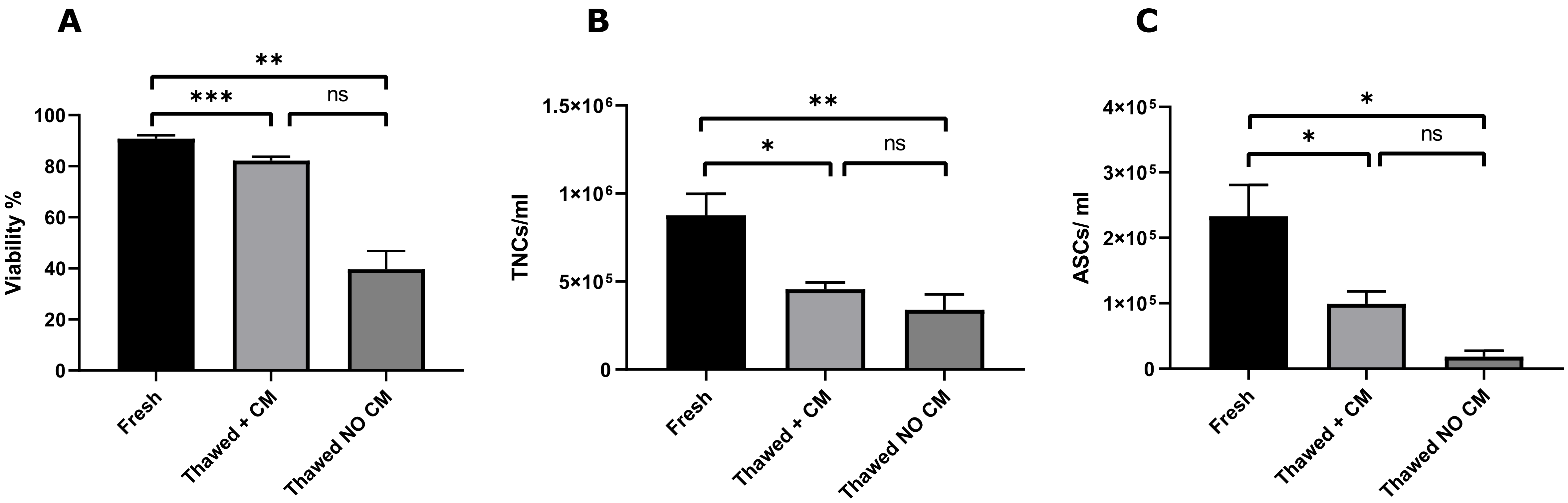

Figure S2. Comparison of biological parameters between three different conditions in non-GMP samples. A) Cell viability %; B) TNCs/ml; C) ASCs/ml. Fresh samples N=14; Thawed + CM N=14; Thawed no CM N= 4.
*p<0.05; ** p< 0.01; **** p<0.0001.

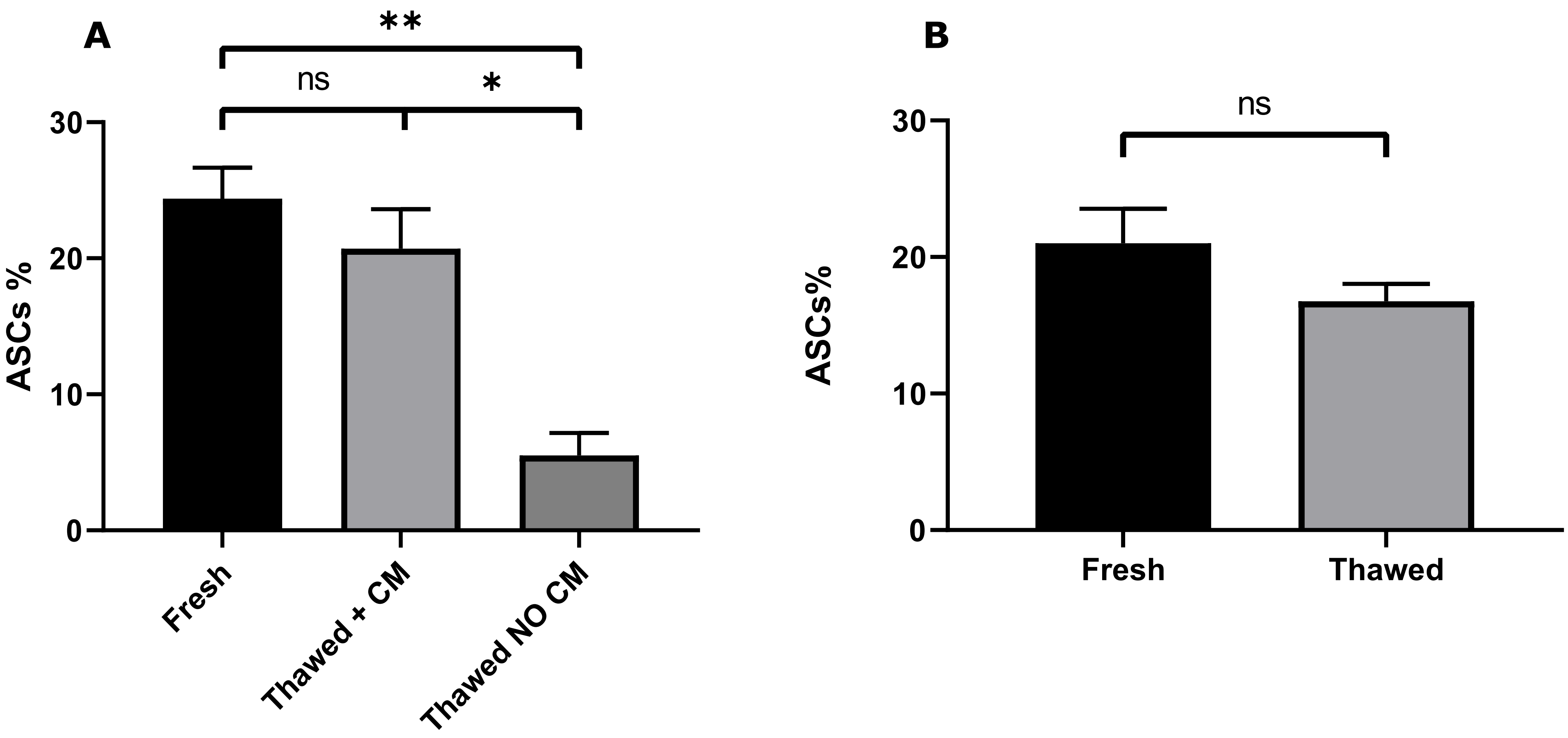

Figure S3. Comparison of ASCs % among Fresh and Thawed samples in non-GMP and GMP samples. A) non-GMP samples N= 14 for fresh and Thawed + CM, N=4 thawed NO CM; B) GMP Samples N= 5. * $p<0.05$; ** $p<0{,}01$; ns= not significative

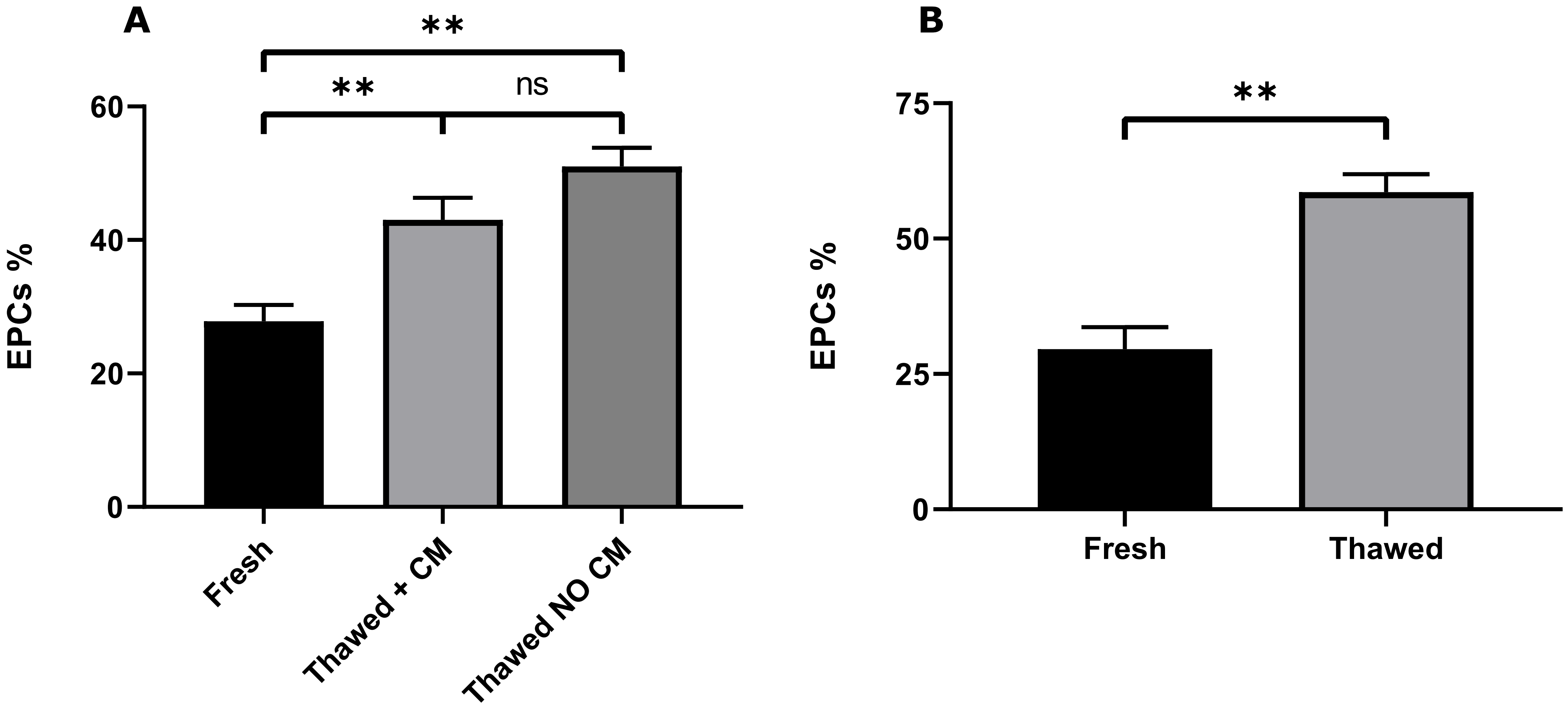

Figure S4. Comparison of EPCs % among Fresh and Thawed samples in non-GMP and GMP samples. A) non-GMP samples N= 14 for fresh and Thawed + CM, N=4 thawed NO CM; B) GMP Samples N= 5. ** $p< 0.01$; ns= not significative.

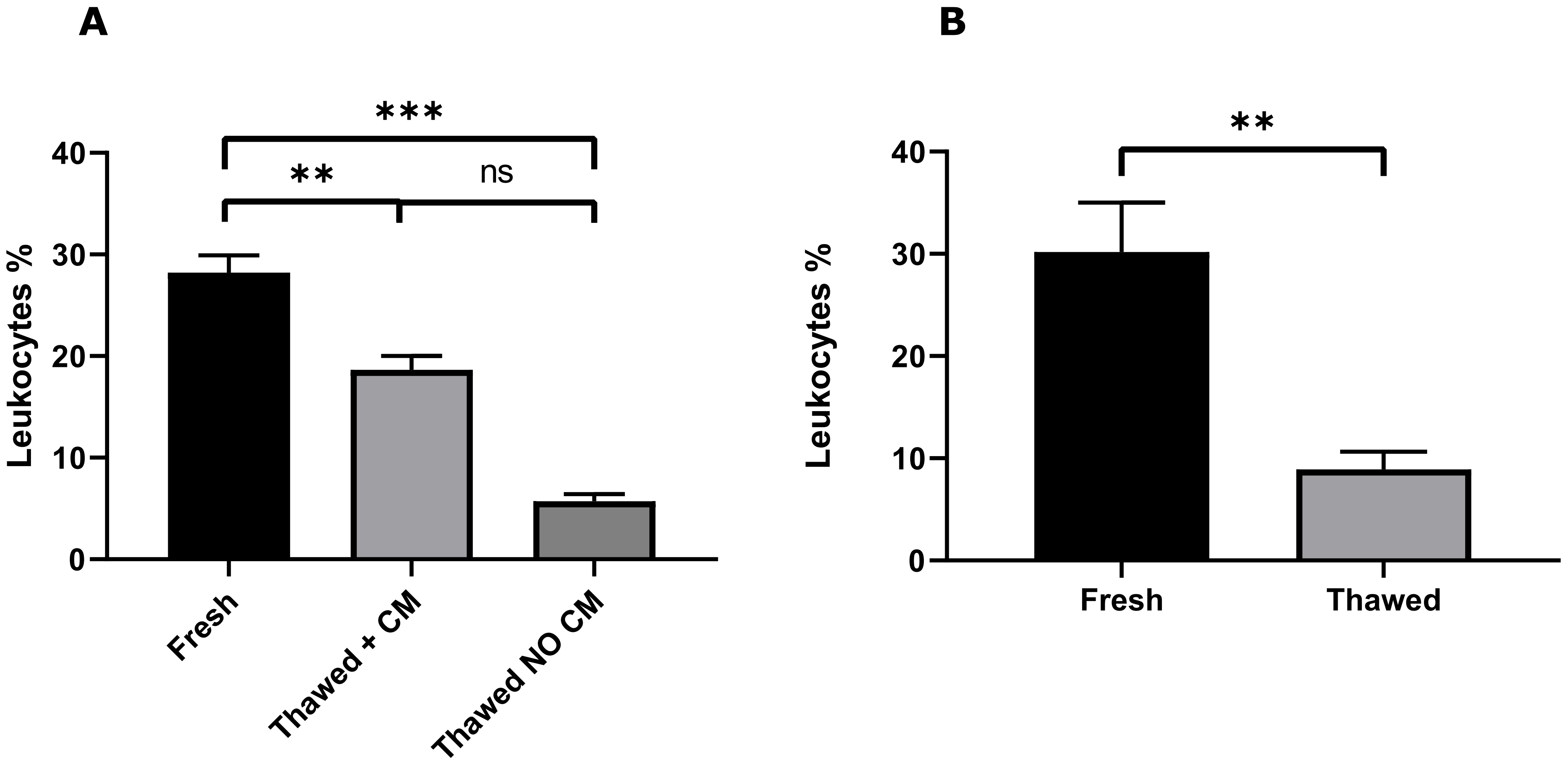

Figure S5. Comparison of Leukocytes % among Fresh and Thawed in non-GMP and GMP samples. A) non-GMP samples N= 14 for fresh and Thawed + CM, N=4 thawed NO CM. B) GMP Samples N= 5. ** p< 0.01; ***p< 0.001.

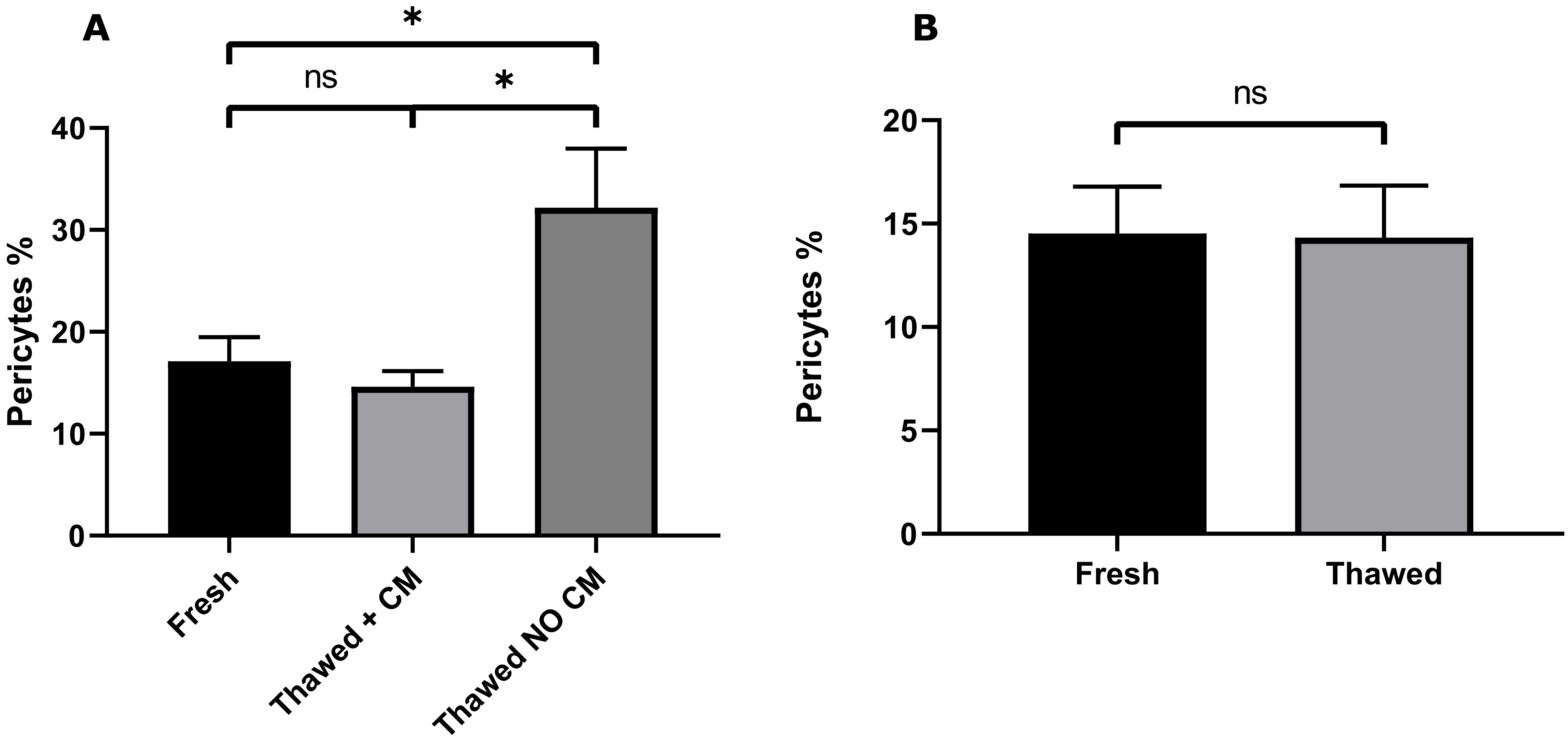

Figure S6. Comparison of Pericytes % among Fresh and Thawed in non-GMP and GMP samples. A) non-GMP samples N= 14 for fresh and Thawed + CM, N=4 thawed NO CM; B) GMP Samples N= 5. * $p<0.05$; ns= not significative, ≥ 0.05.
